# Supplementary material for: Hyperprogression Under Immune Checkpoint-Based Immunotherapy—Current Understanding, The Role of PD-1/PD-L1 Tumour-Intrinsic Signalling, Future Directions and a Potential Large Animal Model
Source: Cancers (Basel). 2020 Mar 27;12(4):804. doi: 10.3390/cancers12040804 (PMC7226013; doi:10.3390/cancers12040804)
Supplement: Supplementary file 1 [file cancers-12-00804-s001.pdf]

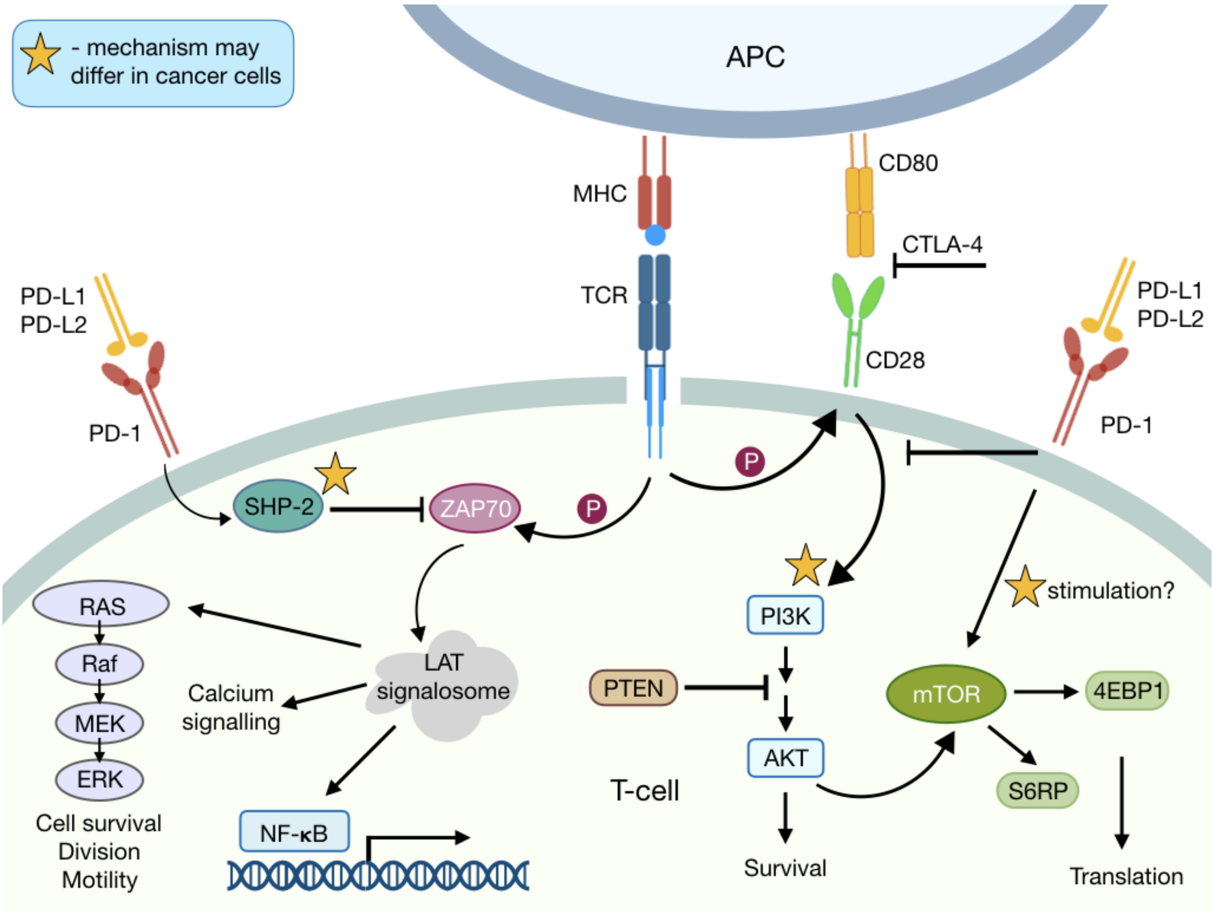

Figure S1. Mechanisms underlying T-cell activation with potential differences in PD-1/PD-L1 tumour intrinsic signaling. T-cell activation is a two-step process that begins with TCR recognition of an antigen loaded on MHC localized on APC. Only TCR specific antigens induce further signaling. T-cell activation can only be completed by costimulatory - antigen independent - stimulus. Despite numerous T-cell co-activators, the interaction between CD80/86 molecules of APC and CD28 protein of the T-cell is considered as the most significant. TCR signaling triggers a cascade of downstream events mediated by ZAP70 phosphorylation, followed by LAT signalosome formation. It is essential for signal propagation to calcium-dependent pathway, the mitogen- activated protein kinase (MAPK) and to the nuclear factor κB (NF- κB) pathway. These pathways lead to the recruitment of transcription factors to chromatin and gene expression fundamental for T-cell proliferation, differentiation, motility or stress response. Alternatively, TCR signaling acts through PI3K activated by the co-stimulatory interaction between CD28 and CD80/86. PI3K signal transduction leads to AKT stimulation that is controlled by PTEN negative regulation. AKT activity facilitates mTOR signal propagation by either of its downstream effectors: S6RP or 4EBP1 as their activity is coupled with recruitment of ribosomes to mRNA and mRNAs translation. T-cell activation is negatively controlled by CTLA-4 and PD-1 immune-checkpoint activity. While CTLA-4 disrupts co-stimulatory signaling competing with CD28 for binding to CD80/86 molecule, PD-1 signaling negatively regulates activated T-cell function. Upon PD-1 engagement by PD-L1, SHP-2 is recruited to PD-1 cytoplasmic tail to further abrogate ZAP70 and ERK signaling as well as CD28 mediated activation of PI3K - a key mediator for complex AKT signaling. Consequently, mTOR pathway is no longer stimulated leading to impairment of cellular growth, proliferation and survival and ultimately to T-cell functional exhaustion. * The star symbol was used to emphasize mechanisms that may be differently regulated in PD-1/PD-L1 tumour intrinsic signaling and were extensively discussed in the main text of this review.
